# Joint motion of bipolar hemiarthroplasty in routine hip functional movements: a dynamic motion study

**DOI:** 10.1186/s12891-020-03749-6

**Published:** 2020-11-10

**Authors:** Weizhou Jiang, Jun Xiao, Bin Chen, Ming Jia, Yang Zhang, Jian Wang, Zhanjun Shi

**Affiliations:** 1 Department of Orthopaedics, Nanfang Hospital, Southern Medical University, Guangzhou, 510515 China; 2 Department of Radiology, Nanfang Hospital, Southern Medical University, Guangzhou, 510515 China

**Keywords:** Bipolar hemiarthroplasty, Motion, Inner bearing, Outer bearing, Treadmill, Osteoarthritis, Femoral neck fractures

## Abstract

**Background:**

Many motion studies have shown that the inner bearing of bipolar prostheses moves less than expected under non-weight-bearing and static weight-bearing positions, which are not routine functional movements performed postoperatively. The aim of this study was to investigate the behaviours of bipolar prostheses during normal gait and simulative squatting.

**Methods:**

Thirty-one femoral neck fracture patients were enrolled, and fluoroscopy examinations of walking on a treadmill, simulative squatting, and non-weight-bearing abduction-adduction and flexion-extension motions were performed at an average of 40 months postoperatively. The rate of acetabular cartilage degeneration was calculated. The ranges of motion of the outer bearing and inner bearing were determined, and the O/I ratios were calculated. Clinical efficacy was assessed by HHS and EQ-5D score.

**Results:**

The inner bearing moved more than the outer bearing did, with an O/I ratio of 0.81, during the normal gait examination, while the motion of the outer bearing was obviously dominant during the simulative squatting and non-weight-bearing abduction-adduction and flexion-extension examinations. The mean acetabular cartilage degeneration rate was 0.82 ± 0.54 mm/year at the follow-up. In subgroup analyses, the motion of the outer bearing decreased to some extent with the increase in acetabular wear, and the corresponding O/I ratios among the groups showed a trend of decreasing first and then increasing. The HHS and EQ-5D scores of the patients with osteolysis and femoral stem loosening were much worse than those with fixed implants.

**Conclusion:**

Bipolar prostheses do function as originally intended during gait, but movement primarily occurs at the outer bearing during other examinations. The motion patterns of bipolar prostheses change with the increase in acetabular wear.

## Background

Bipolar hemiarthroplasty (HA) was initially developed for the treatment of displaced femoral neck fractures in elderly people to reduce the complications of unipolar HA, such as acetabular erosion and groin pain, which are caused by movement between the hard metal head and the acetabular cartilage [1, 2]. Bipolar prostheses are designed to have two articulations. The inner, low friction bearing is composed of a metal inner head and a polyethylene liner, while the outer bearing is composed of a metal outer head and an acetabulum. Theoretically, movement should mainly occur at the inner bearing when the hip is moving in a small range, and the polyethylene liner should absorb some impact stress of the hip during weight-bearing; thus, acetabular erosion may be delayed or avoided.

However, some studies have casted doubt on the theoretical benefits of the bipolar system. It has been found that the inner bearing loses mobility and becomes stiff quickly, and in most cases, movement only occurs at the outer bearing, with the prothesis serving as a unipolar unit, during various non-weight-bearing and static weight-bearing positions [3, 4]. Bipolar prostheses have been found to behave as originally expected, mainly in patients with osteoarthritis and rheumatoid arthritis [5, 6]. However, considering the high risk of osteolysis and femoral stem loosening during the long-term follow-up in osteoarthritis patients, [7, 8] bipolar HA has been mainly performed in elderly patients with hip fractures.

It is noticeable that most of the radiographic motion studies previously conducted focused on non-weight-bearing and static weight-bearing positions and did not investigate routine functional movements, such as walking and squatting [3–6, 9–13]. We therefore performed this study to investigate the behaviours of bipolar prostheses during normal gait and simulative squat motion using video radiography, aiming to assess the motion of the inner and outer bearing surfaces of a bipolar system under various real-life settings.

## Methods

### Participants

Between January 2010 and June 2018, 189 patients (mean age at the time of surgery: 79.34 ± 8.07 years, range from 50 to 97 years) with femoral neck fractures underwent unilateral bipolar HA in our institution. Patients with pathological fractures, osteoarthritis, rheumatoid arthritis, a history of arthroplasty and a history of revision for other surgical treatments were excluded. Of the patients included, 99 were deceased before the follow-up, 38 were unable or unwilling to participate in the follow-up examinations because of old age or age-related medical and domestic problems, 8 had cognitive impairments, 9 had a history of stroke and 4 had undergone revision surgery due to dislocation or periprosthetic fractures. Finally, 31 patients (31 hips) were enrolled in this study and completed all the follow-up examinations in January 2019. This study is a retrospective study of prospectively collected data. Information on the preoperative diagnosis, surgical intervention and postoperative X-ray examination were obtained from the medical records. Written informed consent was provided by each patient, and this study was reviewed and approved by the institutional research ethics committee of Nanfang Hospital, Southern Medical University.

### Surgical intervention

All the procedures were performed by the same group of experienced orthopaedic surgeons through a posterolateral approach. Reaming of the acetabulum was not performed. The bipolar prostheses used in this study were as follows: UHR™ universal head bipolar system (Stryker Orthopaedics™, USA) in 20 patients and Tandem™ bipolar system (Smith & Nephew™, USA) in 11 patients. All stems were uncemented. The diameter of the outer head was determined by measuring the diameter of the femoral head.

### Clinical evaluation

The clinical evaluation included the Harris Hip Score (HHS) for the assessment of hip function and the EuroQol five-dimensional questionnaire (EQ-5D) for the assessment of health-related quality of life (HRQoL). The final HHS scores were graded as excellent (> 90), good (80–89), fair (70–79) or poor (< 69). An EQ-5D score of 0 indicated the worst possible health status, and a value of 1 indicated the best possible health status.

### Radiographic evaluation

For each patient, standard radiographs in the anteroposterior (AP) view of the pelvis were taken within the first 2 days postoperatively and at the follow-up. Osteolysis was defined as localized bone resorption or endosteal erosion, and acetabular protrusion was defined as the invasion of the bipolar cup over Kohler’s line [14]. Loosening of the femoral stem was defined as a radiolucent line of width over 2 mm occupying the entire surface of the implant [15]. Implants without any of the evidence mentioned above were defined as stable and fixed implants. Each patient was evaluated by two radiologists. The evaluation results were confirmed only when both radiologists agreed on the results; otherwise, re-evaluations were performed to determine the final evaluation results. The method described by Muraki et al. [16] was applied to assess the distance of migration of the bipolar head. The rate of acetabular cartilage degeneration was calculated by dividing the distance of migration of the bipolar head by the length of the follow-up period.

For the motion study, the following fluoroscopy examinations were conducted.

#### Non-weight-bearing abduction-adduction

The patients were supine on the X-ray table with the pelvis fixed and the involved leg neutrally positioned. Then, the patients were asked to move the leg from maximum abduction to maximum adduction, and fluoroscopic video imaging was performed in the coronal plane.

#### Non-weight-bearing flexion-extension

The patients were placed in a lateral position, with the horizontal axis of the body perpendicular to the X-ray table. Then, the patients were asked to move the leg from the neutral extension position to maximum flexion and then back to neutral extension; at the same time, fluoroscopic video imaging was performed in the sagittal plane.

#### Weight-bearing simulative squat motion

**Fig. 1:**
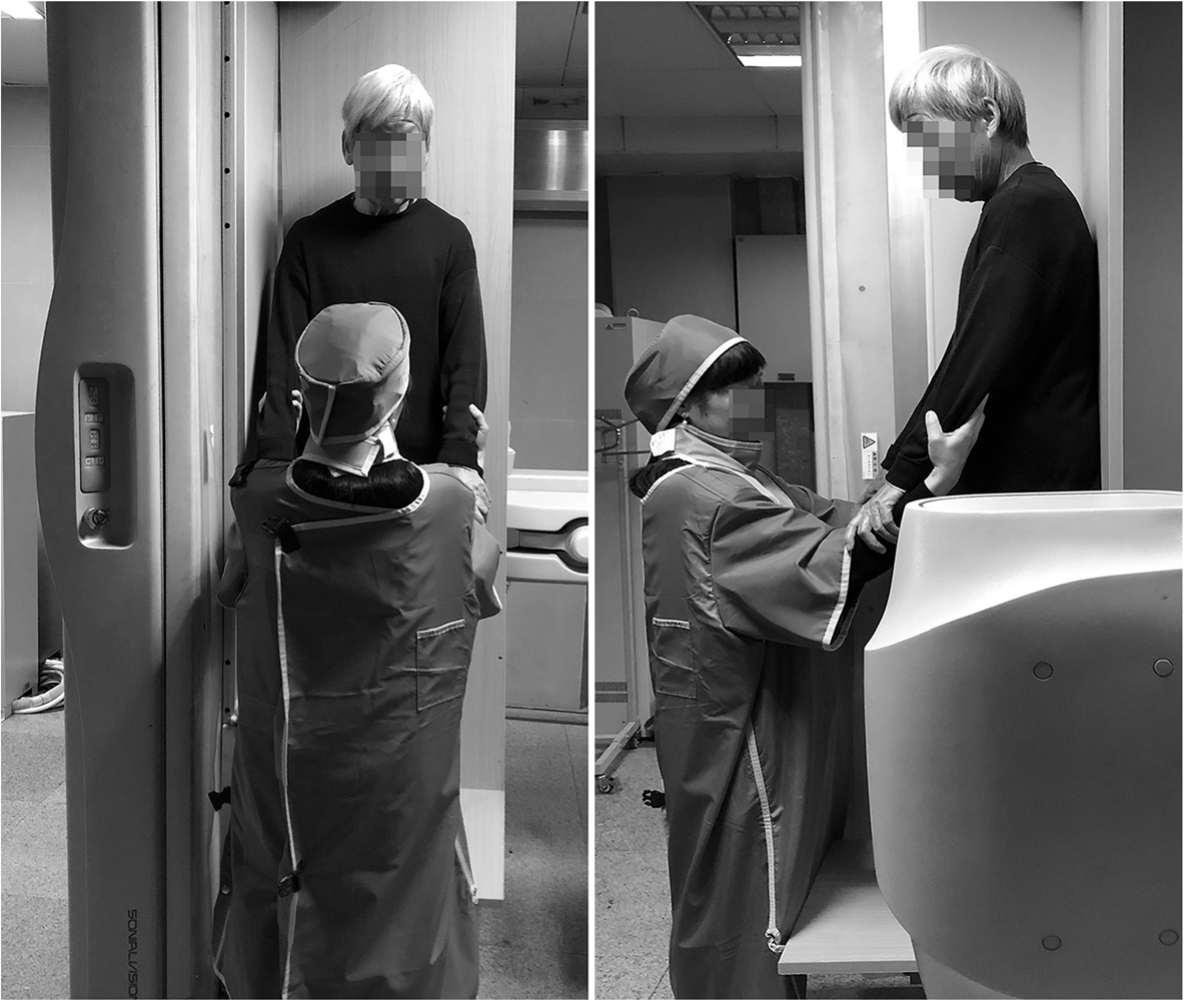
Weight-bearing simulative squat motion examination**.** The patients were requested to stand on the examination board with their buttocks and heels in contact with the board, which was perpendicular to the ground. Then, the patients were asked to squat down slowly and then return to the upright posture. The speed of these movements matched the speed of the X-ray tube, and fluoroscopic video imaging was performed in the sagittal plane

The patients were requested to complete the examination, as shown in Fig. 1.

#### Weight-bearing normal gait motion

**Fig. 2:**
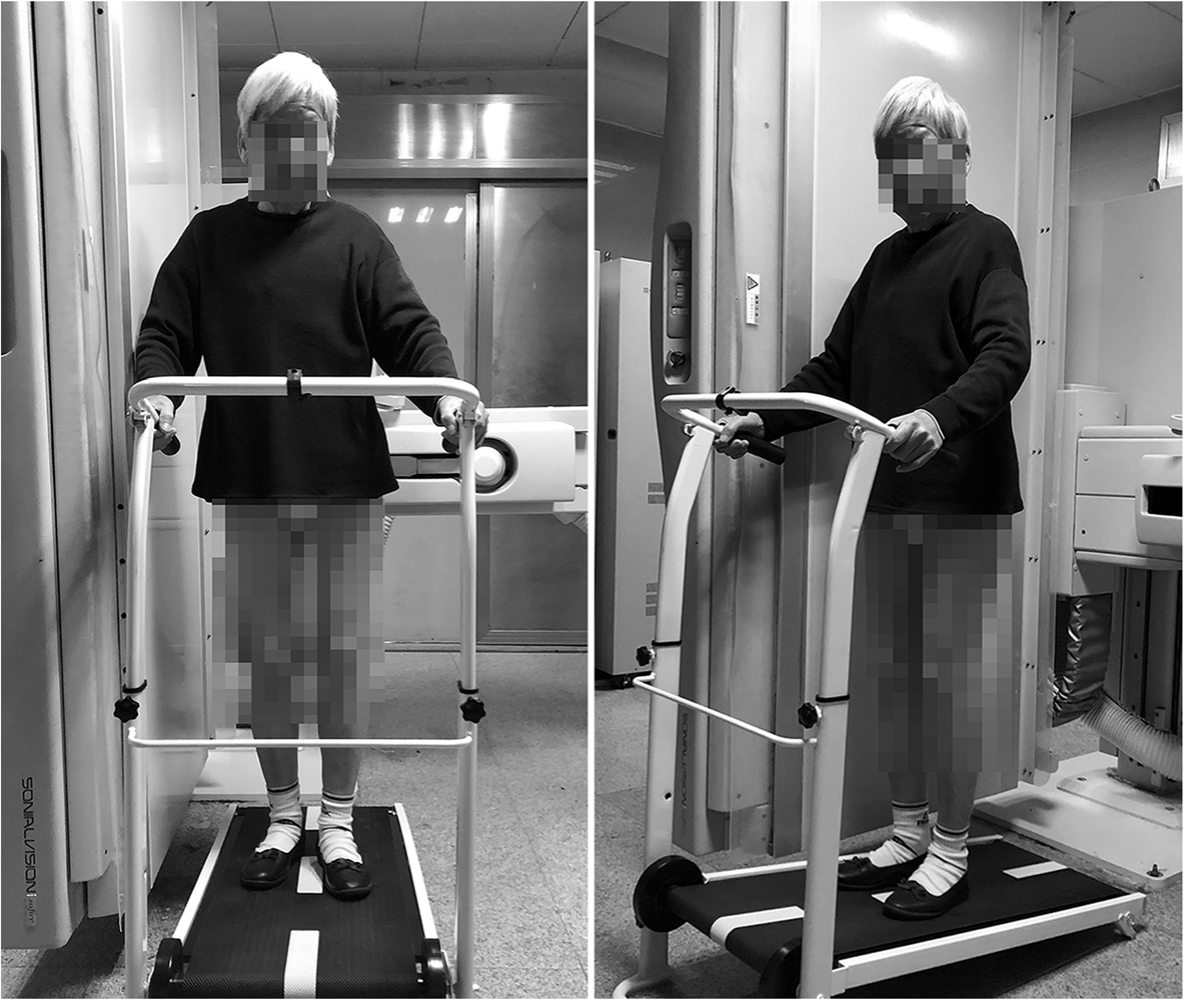
Weight-bearing normal gait motion examination. The patients were asked to stand on the treadmill and then to walk on it at a normal pace. Fluoroscopic video imaging was conducted in the sagittal plane

The patients were requested to complete the examination, as shown in Fig. 2. The treadmill used in this study was not electronic; therefore, a certain slope was required for the belt to operate properly. We did not elevate the backside of the treadmill during the examination for safety reasons.

**Fig. 3:**
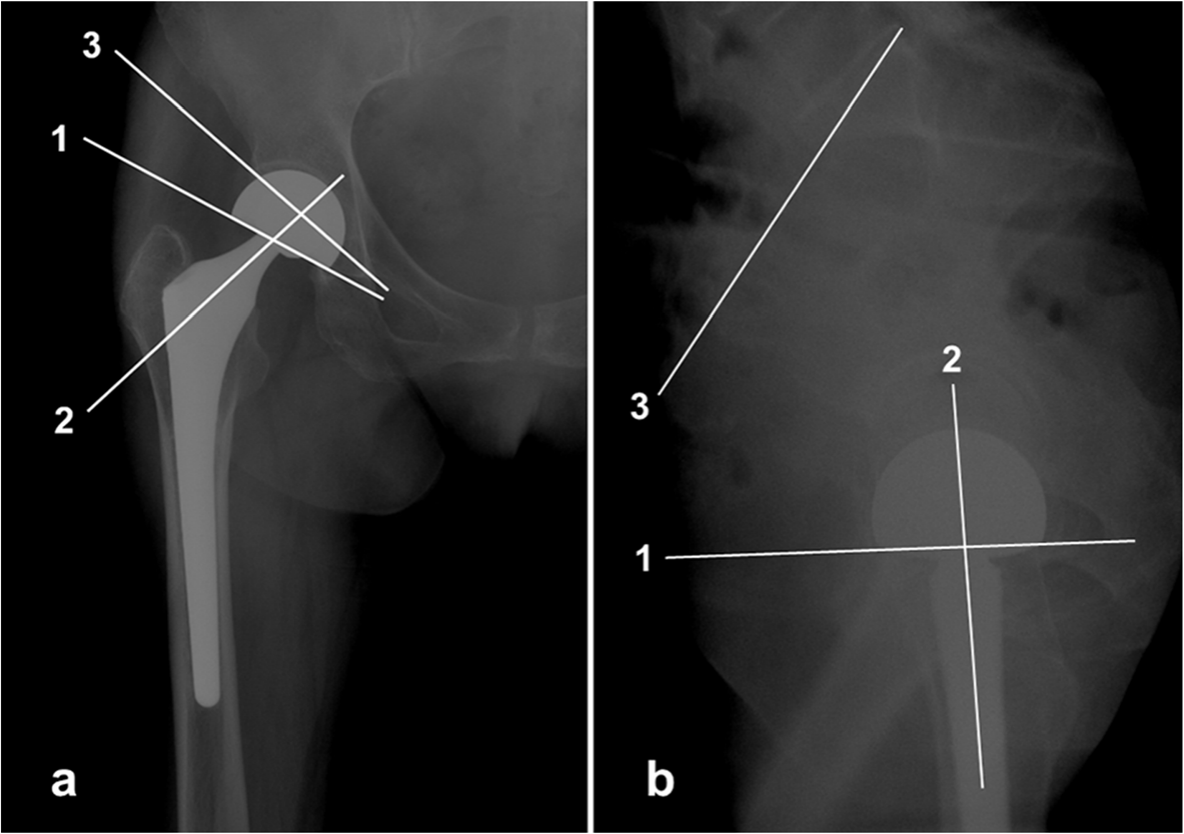
Measurements of the degree of joint motion of the bipolar prosthesis. According to the method described by Drinker and Murray (1979), the anteroposterior view was used to evaluate the abduction-adduction motion (**a**) and the lateral view was used to evaluate the flexion-extension motion (**b**): line 1 is parallel to the straight lateral edge of the outer cup, line 2 passes through the middle of the neck of the prosthesis, and line 3 is the acetabular index in the anteroposterior view and an extension of the upper margin of the sacrum in the lateral view. Angle 1–2 = inner motion; angle 1–3 = outer motion; angle 2–3 = total motion

To determine the degree of total motion, inner motion and outer motion of each bipolar prosthesis, we used the method described by Drinker and Murray [11] (Fig. 3). The ratio of the outer/inner bearing motions (O/I ratio) were calculated. The angles of motion of the bipolar protheses were measured over four or five cycles of non-weight-bearing abduction-adduction, flexion-extension, weight-bearing simulative squatting and walking. The fluoroscopy examinations were performed at a frequency of eight frames per second. Consecutive still frames were analysed to quantify the relative motion of the prosthetic bearings.

To investigate the influence of different acetabular statuses on the motion pattern of the bipolar protheses, thirty-one enrolled patients were divided into four groups according to the distance of migration of the bipolar head (Group A: 0–1 mm; Group B: 1–2 mm; Group C: 2–3 mm; Group D: > 3 mm). To assess the association between the imaging outcomes and clinical outcomes, thirty-one enrolled patients were further divided into three groups according to the status of the implants (osteolysis group, loosening group and fixed group) at the time of the follow-up.

### Statistical analysis

The data were analysed using SPSS software, v19.0 (IBM, NY, USA). The measurement data are presented as the mean ± standard deviation (SD). The patients were divided into four groups according to the distance of migration of the bipolar head, as described above. Subgroup analyses of the motion indexes were performed with the Mann-Whitney U test. A *P* value< 0.05 was identified as statistically significant.

## Results

### Demographics

The general characteristics of the 31 enrolled patients are presented in Table 1. At the follow-up, the mean HHS score of all the enrolled patients was 77.93 ± 15.90 (excellent 9, good 6, fair 7, poor 9). Osteolysis was found in 6 patients, loosening of the femoral stem was found in 5 patients, no cases of acetabular protrusion were found, and the implants of the remaining 20 patients were confirmed to be stable and fixed. The mean distance of migration of the bipolar head was 2.26 ± 1.52 mm and the mean rate of acetabular cartilage degeneration was 0.82 ± 0.54 mm/year at the follow-up.

**Table 1:** General characteristics of the enrolled patients

Patient characteristics	(*n* = 31)
Males, n (%)	10 (32.3%)
Age ^a^ (y): mean (SD); range	78.84 (6.02); 67 to 91
Follow-up time (mo): mean (SD); range	39.74 (26.38); 7 to 105
HHS score ^b^: mean (SD); range	77.93 ± 15.90 (45.23–98.94)
EQ-5D score ^b^: mean (SD); range	0.72 ± 0.10 (0.532–0.848)
Osteolysis ^b^, n (%)	6 (19.4%)
Femoral stem loosening ^b^, n (%)	5 (16.1%)
Fixed implant ^b^, n (%)	20 (64.5%)
Migration of the bipolar head ^b^ (mm): mean (SD); range	2.26 (1.52); 0.38 to 6.45

*Abbreviation*: *n* number of cases, *y* years, *SD* standard deviation, *mo* month, *mm* millimetres, *HHS* Harris Hip Score, *EQ-5D* EuroQol five-dimensional questionnaire

^a^ at the time of surgery

^b^ at the time of the follow-up

### Radiographic motion examination

As shown in Table 2, the mean degree of motion of the outer bearing was 30.03, while that of the inner bearing was 4.75, with an O/I ratio of 6.32 during non-weight-bearing abduction-adduction movement. In addition, similar motion patterns were also detected during non-weight-bearing flexion-extension (O/I = 4.59) and the weight-bearing simulative squat motion (O/I = 7.11). Nevertheless, it was found that the mean degree of motion of the outer bearing was 17.64, while that of the inner bearing was 21.74, with an O/I ratio of 0.81 during normal gait.

**Table 2:** Outcomes of the motion indexes during 4 different motion examinations

	Amount of Motion (degrees)			
	Total, mean (SD)	At the Outer Bearing, mean (SD)	At the Inner Bearing, mean (SD)	O/I Ratio
**Abduction-adduction**	34.78 (9.64)	30.03 (9.40)	4.75 (4.02)	6.32
**Flexion-extension**	69.86 (12.49)	57.36 (14.41)	12.50 (12.25)	4.59
**Simulative squat**	50.18 (13.75)	43.99 (16.34)	6.19 (9.46)	7.11
**Normal gait**	39.39 (8.57)	17.64 (13.39)	21.74 (14.47)	0.81

*Abbreviation*: *O/I Ratio* the ratio of outer/inner bearing motions

### Subgroup analyses

**Fig. 4:**
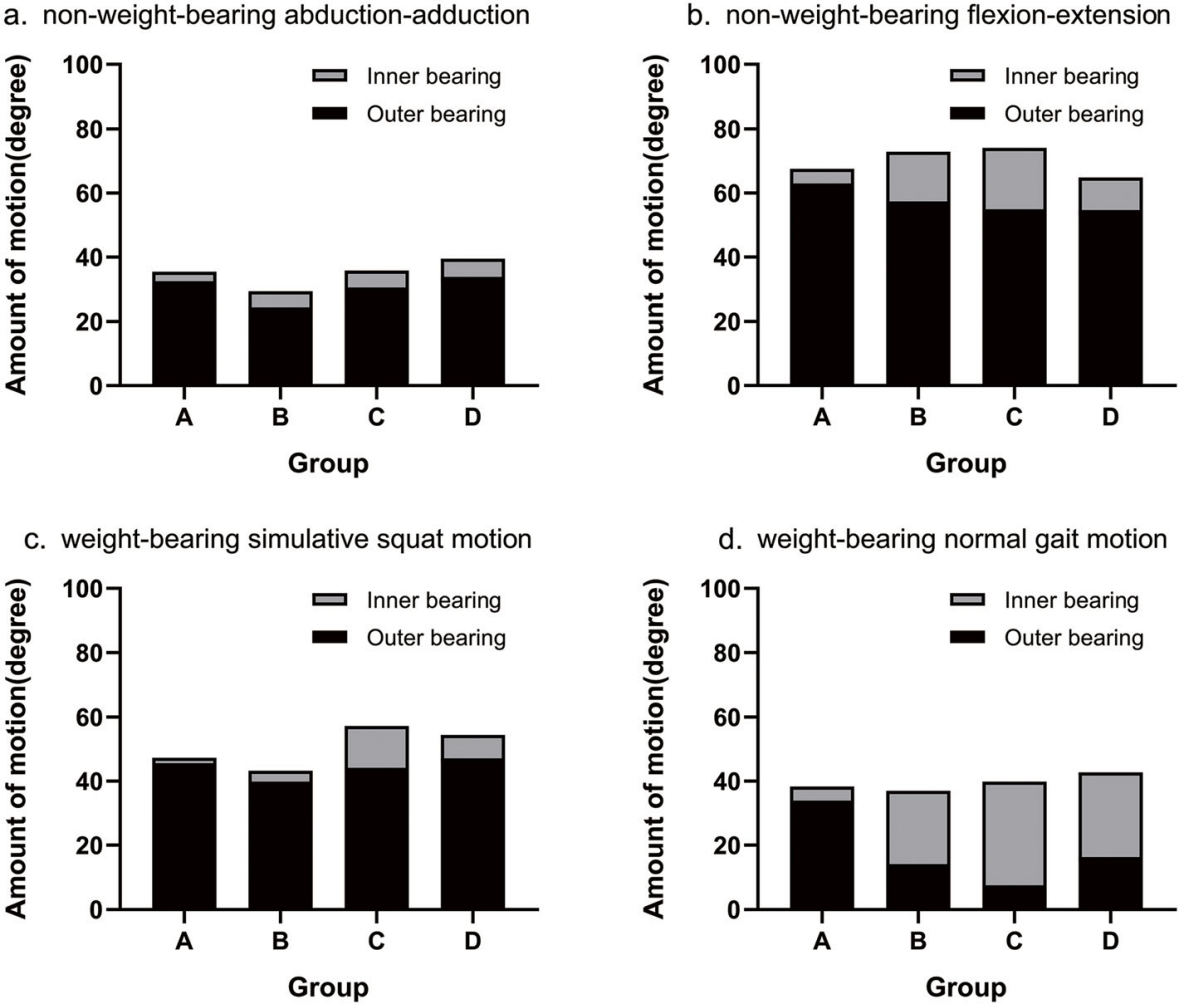
Motion distributions in 4 subgroups during the 4 motion examinations. *: There were no significant differences in the mean total motion among the groups during each specific examination, except that in Group C was larger than that in Group B during the weight-bearing simulative squat examination (57.18° vs. 43.22°, *P* = 0.017). We believe that these differences may be due to individual differences across the enrolled patients. In cases where acetabular protrusion is absent, acetabular wear may change the behaviour of the bipolar prosthesis but may not significantly affect the normal range of motion of the hip. a. During non-weight-bearing abduction-adduction, the degrees of motion of the outer bearing were 32.44, 24.46, 30.49 and 33.79 in Groups A, B, C and D, respectively; the degrees of motion of the inner bearing were 3.03, 4.84, 5.28 and 5.67 in Groups A, B, C and D, respectively. b. During non-weight-bearing flexion-extension, the degrees of motion of the outer bearing were 62.93, 57.35, 54.89 and 54.66 in Groups A, B, C and D, respectively; the degrees of motion of the inner bearing were 4.62, 15.51, 19.18 and 10.16 in Groups A, B, C and D, respectively. c. During the weight-bearing simulative squat motion, the degrees of motion of the outer bearing were 45.59, 39.84, 44.19 and 47.09 in Groups A, B, C and D, respectively; the degrees of motion of the inner bearing were 1.66, 3.38, 12.99 and 7.35 in Groups A, B, C and D, respectively. d. During the weight-bearing normal gait motion, the degrees of motion of the outer bearing were 33.89, 14.09, 7.49 and 16.32 in Groups A, B, C and D, respectively; the degrees of motion of the inner bearing were 4.41, 22.89, 32.31 and 26.36 in Groups A, B, C and D, respectively

**Fig. 5:**
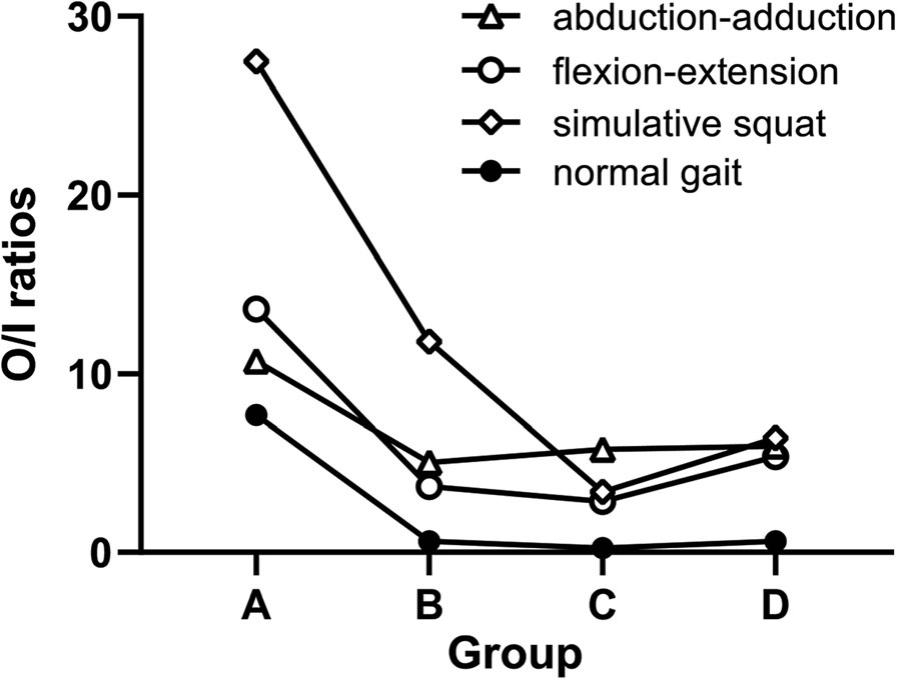
The O/I ratios in 4 subgroups during the 4 motion examinations. During non-weight-bearing abduction-adduction, the O/I ratios were 10.71, 5.05, 5.77 and 5.96 in Groups A, B, C and D, respectively; during non-weight-bearing flexion-extension, the O/I ratios were 13.62, 3.70, 2.87 and 5.38 in Groups A, B, C and D, respectively; during the weight-bearing simulative squat motion, the O/I ratios were 27.46, 11.79, 3.40 and 6.41 in Groups A, B, C and D, respectively; during the weight-bearing normal gait motion, the O/I ratios were 7.68, 0.62, 0.23 and 0.62 in Groups A, B, C and D, respectively. The corresponding O/I ratios among the groups showed a trend of decreasing first and then increasing

Thirty-one enrolled patients were divided into four groups according to the distance of migration of the bipolar head (Group A: 0–1 mm; Group B: 1–2 mm; Group C: 2–3 mm; Group D: > 3 mm) (Fig. 4, Fig. 5). There were seven patients in Group A, with a mean distance of migration of 0.64 ± 0.21 mm (range: 0.38–0.91 mm); nine patients in Group B, with a mean distance of migration of 1.47 ± 0.31 mm (range: 1.03–1.89 mm); seven patients in Group C, with a mean distance of migration of 2.50 ± 0.32 mm (range: 2.02–2.82 mm); and eight patients in Group D, with a mean distance of migration of 4.35 ± 1.08 mm (range: 3.25–6.45 mm).

The mean outer bearing motion in Group A was significantly more than those in Groups B (*P* = 0.005), C (*P* = 0.002) and D (*P* = 0.028) during the gait examination. This finding indicates that the outer bearing motion decreases with the increase of acetabular wear to a certain extent. No significant differences were found in the mean total amounts of motion between the groups during the gait examination. Therefore, the O/I ratio was correspondingly higher in Group A (7.68) than in Groups B, C and D (0.62, 0.23 and 0.62, respectively).

Except for during gait, most of the motion occurred at the outer bearing during the other three examinations. A similar phenomenon was found: the O/I ratios in Group A were larger than those in Groups B, C and D correspondingly. However, the motions of the inner bearing were obviously less than those of the outer bearing during these three examinations. Interestingly, the corresponding O/I ratios among the groups showed a trend of decreasing first and then increasing.

Thirty-one enrolled patients were further divided into three groups according to the status of the implants at the follow-up (Table 3). The mean HHS score of the fixed group was better than those of the osteolysis group (85.33 vs. 73.17) and the loosening group (85.33 vs. 54.08), and the score of the osteolysis group was better than that of the loosening group. For the assessment of HRQoL, the mean EQ-5D score of the fixed group was better than those of the other two groups as well. However, the difference between the osteolysis group and the loosening group was minimal (0.659 vs. 0.670). Regarding the distance of migration of the bipolar head, the implants of the patients in the osteolysis group had the largest magnitude of migration, while those in the loosening group had the smallest magnitude of migration.

**Table 3:** Comparison of efficacy outcomes at the time of the follow-up

	HHS	EQ-5D	Distance of migration (mm)
**Osteolysis group (6)**	73.17 ± 13.19	0.659 ± 0.799	2.76 ± 1.31
**Loosening group (5)**	54.08 ± 7.66	0.670 ± 0.116	1.83 ± 0.79
**Fixed group (20)**	85.33 ± 11.26	0.752 ± 0.085	2.21 ± 1.71

*Abbreviation*: *HHS* Harris Hip Score, *EQ-5D* EuroQol five-dimensional questionnaire, *mm* millimetres

## Discussion

The present study aimed to investigate the motion pattern of bipolar prostheses under various real-life settings. We found that bipolar prostheses do function as originally intended during gait, and their motion patterns change with an increase in acetabular wear. Previous motion studies have shown varied results with regard to the behaviours of bipolar prostheses in vivo [3–6, 9–13]. Currently, it is believed that the movement occurring at the inner bearing is less than expected in most of these cases [3, 4, 6, 9–11, 13]. However, it should be noted that the movements examined in many motion studies are not routine functional movements in elderly people.

The routine functional movements of most elderly people are supposed to be normal walking, sitting and standing, which are dynamic movements that do not need extreme abduction-adduction or flexion-extension motions. Moreover, the hip biomechanics during normal gait and squatting are different from those during non-weight-bearing movements or static simulative movements with weight-bearing. We believe that analysing the behaviours of bipolar prostheses during normal gait and squatting is more important for explaining the short- and long-term clinical outcomes and guiding the clinical application of bipolar HA.

By assessing the fluoroscopic video images of 31 enrolled patients, we found that the mean motion degree of the inner bearing was 21.74 ± 14.47 during normal gait, while that of the outer bearing was 17.64 ± 13.39, with an O/I ratio of 0.81. The motion of the inner bearing was more than that of the outer bearing, which is inconsistent with the findings of previous studies [3, 9, 10, 12, 13]. Moreover, these patients were asked to complete a non-weight-bearing abduction-adduction examination, which has been used in many studies. We found that the mean motion degree of the inner bearing was 4.75 ± 4.02, while that of the outer bearing was 30.03 ± 9.40, with an O/I ratio of 6.32, which is consistent with the findings of previous studies [3, 10, 12]. This finding indicates that the dominant motion occurring at the outer bearing is not the only motion pattern of the bipolar prosthesis in vivo. Considering that gait is one of the main routine functional movements performed by elderly people, bipolar HA can theoretically protect the acetabular cartilage and improve clinical outcomes.

The fluoroscopic video images taken during the other two examinations were also assessed. Gathering all these outcomes in Table 2, we believe that movement of the bipolar prothesis primarily occurs at the outer bearing in most fracture patients, and the inner movement becomes dominant only during normal gait. This finding may explain the similarity in short- and long-term clinical outcomes that have been observed between bipolar HA and unipolar HA in many previous studies [2, 17–21].

Among these 31 enrolled patients, osteoarthritis of the hip was absent before the surgery and reaming of the acetabulum was not performed during the surgery. Hence, the acetabular cartilage remained intact, and the outer bearing surfaces were relatively smooth. Generally, the friction coefficient between the polyethylene liner and the inner head is larger than that between the normal acetabular cartilage and the metal outer head [22]. Hence, movement occurs primarily at the outer bearing during non-weight-bearing abduction-adduction and flexion-extension motion, as we found and previous studies have reported [3, 4, 9–13].

Under loading conditions, the friction coefficient between the inner surfaces increases due to the reduced fluid film in the inner bearing, which is a perfectly matched surface. However, the outer surfaces are not perfectly matched; hence, more fluid is retained to provide fluid-film lubrication [11, 22]. Therefore, more movement occurs at the outer bearing than at the inner bearing during weight-bearing simulative squats, with a higher O/I ratio. Bednar et al. [9] and Drinker et al. [11] also pointed out that the outer bearing motion accounts for a higher proportion during weight-bearing.

In addition to the general results discussed above, we further investigated whether the motion pattern changes over time. The follow-up period varied across individuals in the study population, but no correlation existed between the O/I ratio and the follow-up time (data not shown). This finding may be explained by the motion pattern being primarily determined by the acetabular status, which is largely determined by the postoperative activity level rather than the follow-up time. Therefore, we performed subgroup analyses by dividing the enrolled patients according to the distances of migration of the bipolar head. In this study, the mean rate of acetabular cartilage degeneration was 0.82 ± 0.54 mm/year at the time of follow-up, which was larger than that (0.34 ± 0.35 mm/year) reported by Kim et al. [12]. The reason may be that the average age of the patients enrolled in this study was older (78.84 vs. 45.8), some degree of degeneration of the acetabular cartilage may be present in elderly patients and lead to poor cartilage function; or our average follow-up time was shorter (3.3 years vs. 7.9 years), as the acetabular cartilage is more intact and smooth in the early postoperative period, the movement occurs primarily at the outer bearing, leading to more acetabular cartilage wear.

In the subgroup analyses, as shown in Fig. 5, with an increase in the degree of wear, the corresponding O/I ratios among the groups showed a trend of decreasing first and then increasing, which indicates that the behaviours of bipolar prostheses are not invariable. When the wear of acetabular cartilage is relatively slight, the bipolar prosthesis functions largely as a unipolar device, with movement occurring primarily at the outer bearing, even during the gait examination in Group A. However, the dual-articulation design of the bipolar prosthesis yields a back-up motion pattern. When the acetabular cartilage was worn to a certain extent, the motion of the inner bearing increased gradually. The O/I ratios in Groups B, C and D were correspondingly lower than those in Group A for all the examinations. In addition, during the gait examination, we observed that movement occurred primarily at the inner bearing in Groups B, C and D. The bipolar prosthesis behaved as originally intended at this stage, alleviating acetabular cartilage wear because the movement at the outer bearing was replaced partly by that at the inner bearing.

Then, an increasing trend in the O/I ratios for these four examinations emerged among the groups. It can be assumed that the outer metal head would continuously wear down the acetabular cartilage when a bipolar prosthesis is used, and the increase of the inner bearing motion mentioned above leads to the wear of the polyethylene liner and the formation of polyethylene debris, which further increases the friction coefficient at the inner bearing and results in a relative decrease in the inner bearing motion afterwards.

This phenomenon further compromises the postoperative clinical outcomes of bipolar HA. The inner bearing motion leads to the formation of polyethylene debris, which increases the risk of osteolysis around the prosthesis and femoral stem loosening, as reported in previous long-term follow-up studies [7, 8, 23]. During the mean follow-up period of 40 months, among the patients enrolled in our study, osteolysis was found in six patients (19.4%), and femoral stem loosening was found in five patients (16.1%); the HHS and EQ-5D scores of these patients were much worse than those of the patients with fixed implants, as shown in Table 3. Intriguingly, we found that patients in the loosening group had the worst HHS score but the minimum distance of migration of the bipolar head. This may be because patients with a loosening prosthesis have relatively poor hip function and suffer more severe pain during movement, which makes these patients unwilling to move. Considering that the revision surgeries of these failed cases are much more difficult and fraught with high complication and loosening rates, [24, 25] we believe that the indications of bipolar HA should still be cautiously limited to hip fractures in elderly patients with a short life expectancy and low activity demand. The integrity of acetabular cartilage should be preserved as much as possible during the surgeries.

This study has several limitations. First, the average age at the time of surgery was 79.34 years old in our institution due to the relatively strict indications of bipolar HA, resulting in a limited number of recruited patients at the follow-up. Second, we kept patients’ hips moving in the same vertical plane during the squat examination because the X-ray tube can only move in a vertical direction. Hence, the measured outcomes of the squats performed in the study may be different from those of normal squats.

## Conclusion

The outcomes of this study show that the bipolar prostheses do behave as their expected based on their design during normal gait and can theoretically protect the acetabular cartilage. The motion patterns of bipolar protheses change with increasing acetabular wear. Considering the effect on the clinical outcomes, further research is needed to provide a better understanding of the motion pattern of bipolar protheses in vivo.

## Data Availability

The data and materials in the study are available from the corresponding author upon reasonable request.

## Abbreviations

HA: Hemiarthroplasty
AP: Anteroposterior
O/I ratio: The ratios of outer/inner bearing motions
SD: Standard deviation
HHS: Harris Hip Score
EQ-5D: EuroQol five-dimensional questionnaire
HRQoL: Health-related quality of life

## References

[1] Giliberty RP (1974). A new concept of a bipolar endo-prosthesis. Orthop Rev.

[2] Hedbeck CJ, Blomfeldt R, Lapidus G, Törnkvist H, Ponzer S, Tidermark J (2011). Unipolar hemiarthroplasty versus bipolar hemiarthroplasty in the most elderly patients with displaced femoral neck fractures: a randomised, controlled trial. Int Orthop.

[3] Verberne GH (1983). A femoral head prosthesis with a built-in joint. A radiological study of the movements of the two components. J Bone Joint Surg (Br).

[4] Chen SC, Badrinath K, Pell LH, Mitchell K (1989). The movements of the components of the Hastings bipolar prosthesis. A radiographic study in 65 patients. J Bone Joint Surg (Br).

[5] Phillips TW (1987). The Bateman bipolar femoral head replacement. A fluoroscopic study of movement over a four-year period. J Bone Joint Surg (Br).

[6] Izumi H, Torisu T, Itonaga I, Masumi S (1995). Joint motion of bipolar femoral prostheses. J Arthroplast.

[7] Pellegrini VD, Heiges BA, Brian B, Lehman EB, Davis CM (2006). Minimum ten-year results of primary bipolar hip arthroplasty for degenerative arthritis of the hip. J Bone Joint Surg (Am Vol).

[8] Nakata K, Ohzono K, Masuhara K, Matsui M, Hiroshima K, Ochi T (1997). Acetabular osteolysis and migration in bipolar arthroplasty of the hip: five- to 13-year follow-up study. J Bone Joint Surg (Br).

[9] Bednar JM, Friedenberg ZB, Turner ML (1988). Bipolar femoral endoprosthesis: a study correlating component movement with clinical outcome. J Trauma Acute Care Surg.

[10] Eiskjaer S, Boll K, Gelineck J (1989). Component motion in bipolar cemented hemiarthroplasty. J Orthop Trauma.

[11] Drinker H, Murray WR (1979). The universal proximal femoral endoprosthesis. A short-term comparison with conventional hemiarthroplasty. J Bone Joint Surg (Am Vol).

[12] Kim YS, Kim YH, Choi IY (2012). The cartilage degeneration and joint motion of bipolar hemiarthroplasty. Int Orthop.

[13] Yoshioka T, Okimoto N, Fuse Y, Kawasaki M, Mori T, Sakai A, Majima T (2018). In-vivo postoperative motion analysis of metal and ceramic bipolar hip hemiarthroplasty. J Orthop Sci Official J Japan Orthop Assoc.

[14] Hubbard S (1969). MJ: the measurement of progression in protrusio acetabuli. Am J Roentgenol Radium Therapy, Nucl Med.

[15] Engh CA, Massin P, Suthers KE (1990). Roentgenographic assessment of the biologic fixation of porous-surfaced femoral components. Clin Orthop Relat Res.

[16] Muraki M, Sudo A, Hasegawa M, Fukuda A, Uchida A (2008). Long-term results of bipolar hemiarthroplasty for osteoarthritis of the hip and idiopathic osteonecrosis of the femoral head. J Orthop Sci.

[17] Christian I, Carl-Johan H, Richard B, Gunilla L, Sari P, Anders E (2013). Unipolar hemiarthroplasty versus bipolar hemiarthroplasty in patients with displaced femoral neck fractures: a four-year follow-up of a randomised controlled trial. Int Orthop.

[18] Wathne RA, Koval KJ, Aharonoff GB, Zuckerman JD, Jones DA (1995). Modular unipolar versus bipolar prosthesis: a prospective evaluation of functional outcome after femoral neck fracture. J Orthop Trauma.

[19] Ong BC, Maurer SG, Aharonoff GB, Zuckerman JD, Koval KJ (2002). Unipolar versus bipolar hemiarthroplasty: functional outcome after femoral neck fracture at a minimum of thirty-six months of follow-up. J Orthop Trauma.

[20] Cornell C, Levine D, O'Doherty J, Lyden J (1998). Unipolar versus bipolar hemiarthroplasty for the treatment of femoral neck fractures in the elderly. Clin Orthop Relat Res.

[21] Raia FJ, Chapman CB, Herrera MF, Schweppe MW, Michelsen CB, Rosenwasser MP (2003). Unipolar or bipolar hemiarthroplasty for femoral neck fractures in the elderly?. Clin Orthop Relat Res.

[22] Tsukamoto Y, Mabuchi K, Futami T, Kubotera D (1992). Motion of the bipolar hip prosthesis components. Friction studied in cadavers. Acta Orthop Scand.

[23] Nishii T, Sugano NK, Takaoka K (1995). Bipolar cup design may lead to osteolysis around the uncemented femoral component. Clin Orthop Relat Res.

[24] Sierra RJ, Cabanela ME (2002). Conversion of failed hip hemiarthroplasties after femoral neck fractures. Clin Orthop Relat Res.

[25] Diwanji SR, Kim SK, Seon JK, Sang JP, Yoon TR (2008). Clinical results of conversion Total hip Arthroplasty after failed bipolar Hemiarthroplasty. J Arthroplast.

